# Disruption and selection: the income gradient in mortality among natives and migrants in Sweden

**DOI:** 10.1093/eurpub/ckad051

**Published:** 2023-04-06

**Authors:** Olof Östergren, Johan Rehnberg, Olle Lundberg, Alexander Miething

**Affiliations:** Department of Public Health, Stockholm University, Stockholm, Sweden; Aging Research Center, Karolinska Institutet, Solna, Sweden; Aging Research Center, Karolinska Institutet, Solna, Sweden; Department of Public Health, Stockholm University, Stockholm, Sweden; Department of Public Health, Stockholm University, Stockholm, Sweden

## Abstract

**Background:**

The income gradient in mortality is generated through an interplay between socio-economic processes and health over the life course. International migration entails the displacement of an individual from one context to another and may disrupt these processes. Furthermore, migrants are a selected group that may adopt distinct strategies and face discrimination in the labour market. These factors may have implications for the income gradient in mortality. We investigate whether the income gradient in mortality differs by migrant status and by individual-level factors surrounding the migration event.

**Methods:**

We use administrative register data comprising the total resident population in Sweden aged between 30 and 79 in 2015 (*n* = 5.7 million) and follow them for mortality during 2015–17. We estimate the income gradient in mortality by migrant status, region of origin, age at migration and country of education using locally estimated scatterplot smoothing and Poisson regression.

**Results:**

The income gradient in mortality is less steep among migrants compared with natives. This pattern is driven by lower mortality among migrants at lower levels of income. The gradient is less steep among distant migrants than among close migrants, migrants that arrived as adults compared with children and migrants that received their education in Sweden as opposed to abroad.

**Conclusions:**

Our results are consistent with the notion that income inequalities in mortality are generated through life-course processes that may be disrupted by migration. Data restrictions prevent us from disentangling life-course disruption from selection into migration, discrimination and labour market strategies.

## Introduction

The relationship between income and health is generated through a reflexive relationship between socio-economic and physiological processes that take place over the life course.^1^^,^^2^ Health in early life and the childhood environment shapes early life physical, cognitive and social development, as well as educational performance and choices.^3–5^ These early life determinants in turn shape the conditions under which the individual enters the labour market. Working conditions are important determinants for health and different occupations are associated with different types of health problems.^6^^,^^7^ The final link in the chain of socio-economic attainment is income, which, from a life-course perspective, is the outcome of social determinants such as education and occupation as well as physiological and cognitive abilities.

Lower income can have negative consequences for health,^8–10^ for example through a lack of material resources or increased stress,^11^ and poor health can in turn shape the individual capacity to participate in the labour market.^12^ Because of these reflexive and longitudinal life-course processes, the relationship between income and health has been described as bi-directional.^13–15^ As a result, persons with lower incomes are more likely to be in poor health, and persons with higher incomes, conversely, are more likely to be in better health.

### The income gradient in mortality among international migrants

Given this understanding of health inequalities as a result of interdependent processes over the life course, a disruptive event could potentially alter the relation between income and mortality. International migration is undertaken for diverse reasons including to avoid political persecution or natural disasters, but also to seek better employment opportunities or to reunite with family. Regardless of the circumstances surrounding the migration, life-course processes may be disrupted when the individual migrates. For example, only a limited amount of material resources can be brought from the country of origin. Similarly, resources embedded in social networks in the country of origin are less useful at the destination and educational qualifications may not be recognised or valued lower, for example because of discrimination in the labour market.^16^ Trajectories of socio-economic position and health initiated in the country of origin may therefore not necessarily continue in the destination country. If migration acts as a disruption of the life-course processes that generate socio-economic inequalities in health, this would in turn result in a less pronounced income gradient in mortality among migrants. How abrupt the disruption is, likely depends on the context of the migration and individual-level factors of migrants.

Migration between countries that are geographically and institutionally close may lead to a less dramatic disruption. In particular, migration between countries with agreements of free mobility may represent a less disruptive event. In the case of Sweden, the empirical context of this study, such an agreement was implemented between the Nordic countries in 1954.^17^ Additionally, restrictions on migration from other European Union (EU) member states were relaxed when Sweden joined the EU in 1995. A wider set of countries were included when the EU expanded in 2004, 2007 and in 2013.^18^ Because of the geographical proximity, in combination with generous migration policies, migrants arriving to Sweden from a Nordic or European country may be able to retain social ties, material resources and non-material resources after the move. This is likely more difficult for migrants arriving from outside of Europe, which may experience a comparatively more dramatic disruption.

While the introduction of free mobility agreements lowers the threshold for some migrants, those arriving from outside of Europe are more likely to have managed a difficult and demanding task. International migration can be an expensive, difficult and physically straining process and the migrants that are able to persevere are likely to be a selected group in terms of material and non-material resources, including health. This phenomenon, referred to as ‘the healthy migrant effect’, likely contributes to the overall lower mortality rate observed among migrants relative to native populations in high-income countries, in particular among distant migrants.^19–21^ Further, labour market discrimination is likely more pronounced among distant migrants that arrive from countries that are more institutionally and culturally different.^16^^,^^22^ Distant migrants are also more likely to face discrimination based on skin colour and to face a more significant language barrier.

Some migrants may adopt different strategies in the labour market, which could make them more willing to accept lower wages than natives. Migrants that compare their income to reference groups in the country of origin, as opposed to the country of destination, may consider an improvement in income relative to the origin to partly compensate for a lower relative income position at the destination.^23^^,^^24^ Income relative to the origin may also be especially important to migrants that are ‘target-earners’, who migrate in order to accumulate a certain amount of capital that they intend to invest in the country of origin.^25^^,^^26^ Taken together, the combination of migrant health selection, challenges in the labour market and different labour market strategies, suggests that migrants are likely to be in better health and are more likely to have lower income than natives and that these patterns are likely more pronounced among distant migrants.

### Hypotheses

We suggest that the processes that shape the income gradient in mortality are likely to differ between natives and migrants. Based on previous research, we expect that the income gradient in mortality is less pronounced among migrants compared with natives due to lower mortality among migrants at lower levels of income. We formulate and test a series of hypotheses using administrative Swedish register data.

H1: The income gradient in mortality among migrants is moderated by geographical proximity and cultural likeness of the country of origin.

We expect the income gradient among migrants from the Nordic countries to be similar to that among the Swedish-born population, given the close proximity between the countries. We expect a flatter income gradient among non-European migrants, as well as lower absolute levels of mortality. We expect the income gradient of European migrants to be in-between the Nordic and non-European migrants.

H2: The income gradient in mortality among migrants is moderated by age at arrival.

Migrants that arrive as children experience a large part of their formative years in the country of destination and are therefore likely to experience exposures and outcomes that are more similar to natives compared with migrants that arrive at older ages. Additionally, health selection is likely more pronounced among adult migrants compared with younger migrants and adult migrants are more likely to compare their income to the distribution in their country of origin. We expect a less pronounced income gradient among migrants arriving as adults compared with migrants arriving as children.

H3: The income gradient in mortality is moderated by the country of education.

Migrants that have completed their education before migration may face difficulties in finding a job that matches their educational qualifications. This may lead to a higher proportion of resourceful migrants at lower levels of income compared with natives at similar income levels. We expect that the income gradient in mortality is less pronounced among migrants educated outside of Sweden.

## Methods

We use administrative register data comprising the total resident population in 2015. We set the lower age bound to 30 when individuals are likely to be active in the labour market. We set the upper age bound to 79, since income inequalities in mortality tend to diminish at older ages^27^ and because of a comparatively small number of non-Nordic international migrants in Sweden at older ages. We define income as the average annual disposable household income between 2011 and 2013. To make income comparable across different household compositions, disposable household income was divided by the square root of number of household members. Income deciles were calculated in the entire sample. We exclude migrants that arrived after the start of income measurement. We classify country of birth into Sweden, Nordic, Europe or other (outside of Europe) and age at arrival into three categories: <7, 7–17 and 18 or more years. We use the registered source from which educational information was reported to the educational register to assess if the individual obtained their highest education in Sweden or another country (see Supplementary table S1 for the complete coding).

We follow the population for mortality between 2015 and 2017, right censoring at death. Selective out-migration has been suggested to bias mortality rates in migrant groups.^28^ To improve the comparability of mortality rates between the natives and migrants, we exclude any person that was registered as emigrated before 2017. Our rationale for the selected years is to include our most recent available data, and to follow the population for mortality long enough to observe stable death rates but not so long as to introduce substantial period effects. Our analytical sample comprises a total of 5.7 million individuals and 109 712 deaths (see table 1). The use of data for the purposes of this study was granted by the Swedish Central Ethical Review Board (Dnr Ö 25-2017).

**Table 1:** Descriptive statistics

	Country of birth			
	Sweden	Nordic	Europe	Other
Individuals (*n*)	4 609 185	176 785	82 182	743 321
Deaths (*n*)	93 761	6486	1628	7362
Median income (1000 SEK)	271	237	237	181
SD income (1000 SEK)	144	142	156	117
Age at arrival *n* (%)				
0–6	—	33 115 (19)	8235 (10)	49 932 (7)
7–17	—	32 191 (19)	6963 (9)	78 395 (11)
18+	—	105 437 (62)	65 368 (81)	611 302 (83)
Country of education *n* (%)				
Sweden	—	151 802 (89)	50 385 (62)	445 165 (60)
Other	—	18 940 (11)	30 181 (38)	294 461 (40)

**Figure 1:**
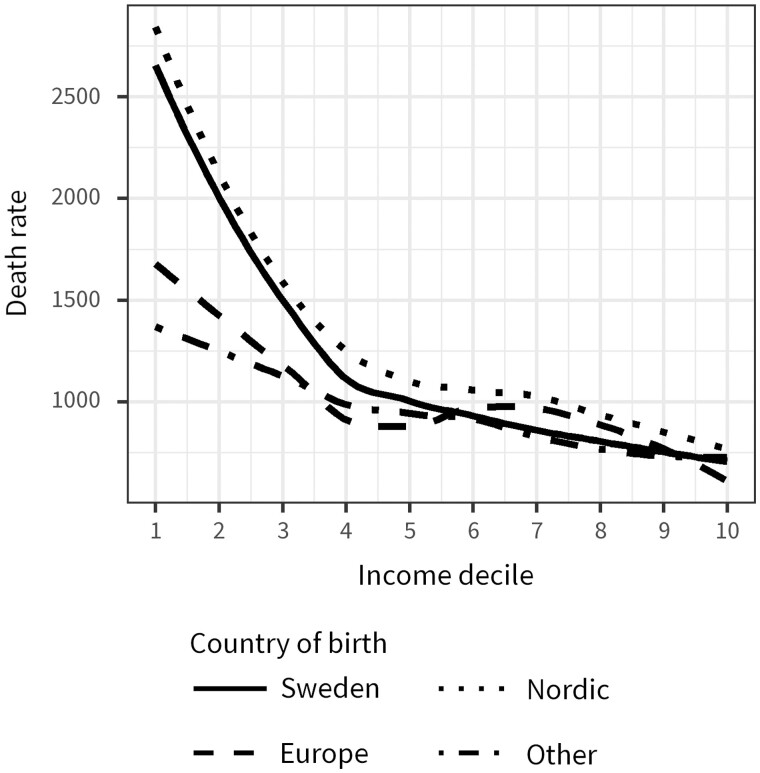
Age-adjusted 3-year death rate per income decile by country of birth among persons aged 30–79 in Sweden, 2015–17. The income deciles are ordered from the bottom incomes (1) to the top incomes (10)

First, we test hypothesis H1 by calculating the age-standardized mortality risk at each income decile relative to the overall distribution of the final sample for natives, Nordic migrants, migrants born in Europe and migrants born outside of Europe. These mortality rates are presented in figure 1 and smoothed over income deciles by using locally estimated scatterplot smoothing (LOESS).The LOESS method is non-parametric and fits multiple regressions that are weighted toward the nearest neighbour observation.^29^ It allows us to visualize the level of mortality and the shape of the income gradient in different migrant groups without making assumptions about the functional form.

Second, we test the hypotheses H2 and H3 using a Poisson regression model defining death as the event and the person-years at risk as the offset. We include covariates for income and country of birth, age at arrival and country of education, including interaction terms between income and each covariate. We model country of birth, age at arrival and country of education in the same model to assess whether these are independent moderators of the association between income and mortality. We also adjust for sex, age and age squared. The interaction terms indicate whether the slope of the income gradient vary by the included covariates. We use the natural logarithm of income to model the log-linear relationship between income and all-cause mortality.^30^ Though this method relies on more assumptions than the mortality rates calculated and presented in figure 1, it allows us to model the curvilinear relationship between income and mortality while maintaining enough degrees of freedom to estimate multiple interaction terms in a single model. The results are visualized by estimating absolute mortality risks based on the coefficients.

## Results

Descriptive statistics are presented in table 1. Migrants have lower incomes than natives. Among migrants, the median income is highest among Nordic migrants and lowest among migrants from outside of Europe. Most migrants arrived as adults, but the proportion of Nordic migrants that arrived as children is higher than among the other two migrant groups. The majority of migrants has obtained their highest educational qualifications in Sweden.

Figure 1 presents the age-adjusted 3-year death rate per income decile by country of birth. The income deciles are ordered from the bottom incomes^1^ to the top incomes.^10^ The income gradient among Nordic migrants is similar to the gradient among natives. In line with our hypothesis (H1), the income gradient is weaker among migrants from Europe and even less pronounced among migrants from outside of Europe. The differences in the income-mortality gradient between the groups are driven by lower levels of mortality among migrants at lower levels of income. Above the fourth income decile the levels of mortality are similar in all groups.

**Figure 2:**
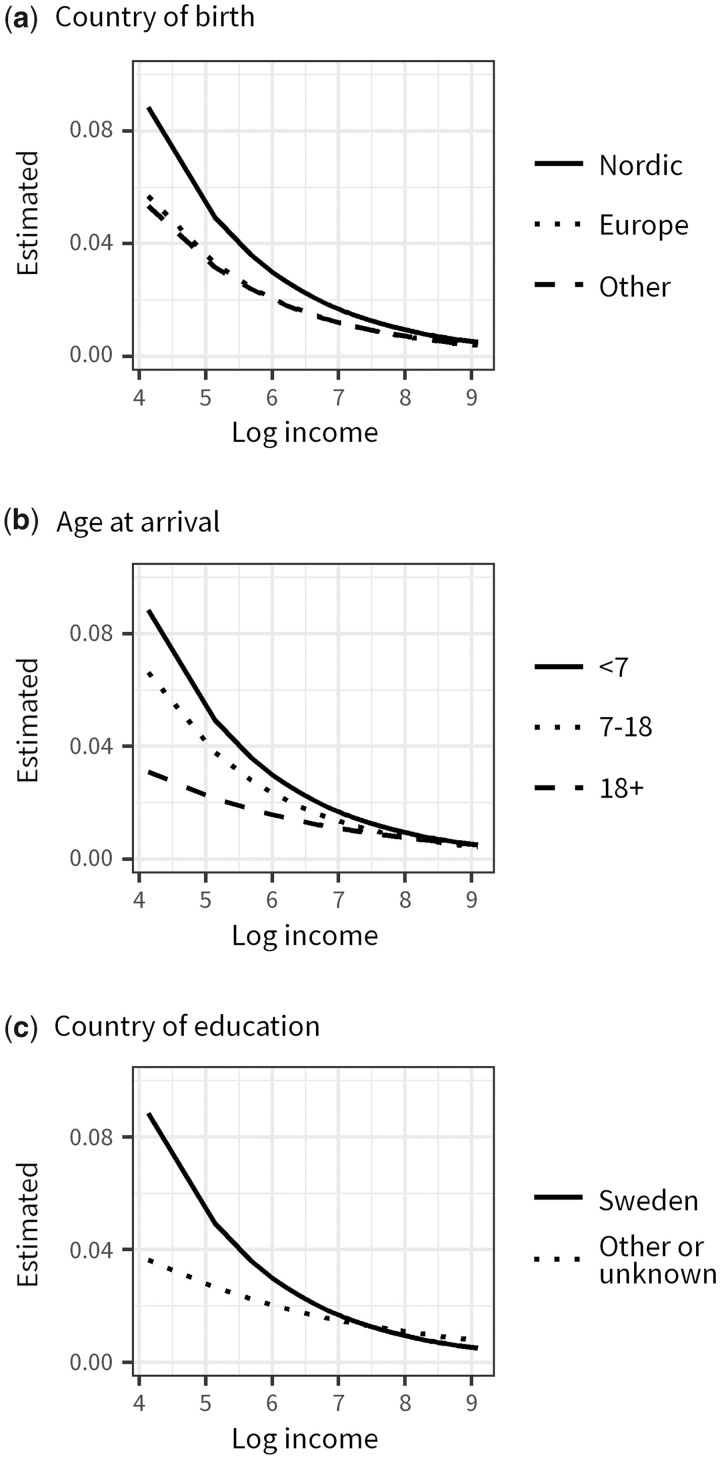
Estimated mortality risk by log income and (a) country of birth, (b) age at arrival and (c) country of education among migrants aged 30–79 in Sweden, 2015–17. *Note*: The mortality risks were estimated from a Poisson regression model where the number of deaths was defined as the dependent variables and person-years at risk as the offset. The model included covariates for income and country of origin, age at arrival, country of education, age and sex as well as interaction terms between income and country of origin, age at arrival and country of education respectively (see Supplementary table S2 for coefficients used to estimate the death risks)

Figure 2 presents mortality risks estimated from a Poisson model. The coefficients are presented in Supplementary table S2. Note that natives are excluded from these analyses. We find similar patterns by country of birthas in the non-parametric results (figure 1). In line with our hypotheses (H2 and H3), we also find systematic variation in the income gradient in mortality by age at migration and country of education, independent of country of birth. The income gradient in mortality is less pronounced among migrants arriving in adult ages, though we find no difference between migrants that arrive as young children and teenagers (figure 2b). The income gradient in mortality was also less pronounced for migrants that obtained their education outside of Sweden (figure 2c).

We additionally estimate separate models for men and women to examine whether sex moderated the income-mortality association. The patterns are similar among men and women, though the absolute level of mortality was lower for women (Supplementary figure S1 and table S3). In order to check for potential multicollinearity, we fit a linear regression model including the main covariates included in our analysis and calculated variance inflation factor (VIF) statistics. These indicate that there are no issues with multicollinearity (Supplementary table S4).

## Discussion

In line with our hypothesis (H1), we find a less pronounced income gradient among migrants compared with natives. In particular, it is less pronounced for distant migrants. We also find variation in the slope of the income gradient in mortality within the migrant group. In line with our hypotheses (H2 and H3), the income gradient in mortality is moderated by age at arrival and country of education. We find weaker income gradients among migrants arriving at a younger age and among migrants that were educated outside of Sweden. The differences in the income-mortality gradients are driven by low mortality rates among migrants with low income. Similar to our findings, Patel et al.^31^ report smaller mortality differences between natives and migrants at higher incomes in Finland. These results indicate that international migration may not only contribute to increasing life expectancy in destination countries but also smaller social inequalities in mortality.

Previous studies have found less pronounced socio-economic gradients in health among foreign-born Hispanics in the USA, relative to the native population.^32^^,^^33^ The focus of these studies has been on the characteristics of the migrants, in particular behavioural risk factors, while focusing less on processes among natives. In contrast, we have argued that the processes that generate health inequalities may differ between natives and migrants. We identified several potential mechanisms that can contribute to a less pronounced income gradient in mortality among migrants. Migration may effectively disrupt the interplay between socio-economic and physiological processes that generate the association between income and mortality. Additionally, the process of migration may place persons in good health at lower incomes through health selection into migration, differences in labour market strategies and discrimination.

The disruption of life-course processes is likely smaller for migrants that arrive from geographically and culturally closer countries, at lower ages and that obtained their educational qualifications in Sweden. However, migrants that arrive from distant countries are likely also more strongly selected on health, more likely to experience discrimination and to differ in their labour market strategies. Based on this study, we cannot disentangle the relative importance of the proposed mechanisms. We found that being born outside of Europe, arriving in adulthood and being educated abroad were independently associated with a less pronounced income gradient. This provides some tentative support for the disruption hypothesis, since country of origin captures both distance and likelihood of discrimination and differences in preference. However, due to data restrictions, we used a broad indicator of country of origin and it is possible that there is residual variation from country of birth that is captured in the age at arrival and country of education.

Discrimination may keep resourceful and healthy individuals from obtaining higher income, though experiences of discrimination may in itself be harmful to health.^34^^,^^35^ For example, migrants in Sweden are more likely to have a higher educational level than what is required for their occupation^36^ which has been found to be associated with lower self-rated health.^37^ On the other hand, other studies have found that the income gradient in mortality is less pronounced at higher levels of education^38^ and it is possible that higher educated migrants with low incomes can to some degree compensate the potentially negative effects that low income has on health. It is therefore not clear if discrimination contributes to a less pronounced income gradient in mortality. Since we are not able to directly observe discrimination in the register data, we are unable to determine the potential influence of discrimination.

### Methodological considerations

Migrants have, on average, lower incomes than natives (table 1). Using the total population to define income deciles allows us to compare mortality levels of migrants and natives that have the same absolute income. However, there may be substantial heterogeneity within deciles for migrants since a larger proportion is concentrated at lower deciles (Supplementary table S5). We therefore excluded the natives, recalculated the income deciles based on the distribution among migrants and plotted the mortality rates (Supplementary figure S2). The results were similar to those presented in figure 1, though we observe lower mortality rates in the first compared with the second decile. It is possible that individuals with low levels of registered income rely on income sources which are not registered,^30^ which introduces some uncertainty around the measurement of income in the low end of the income distribution. Migrants may be more likely to have income sources abroad, but are also more likely to send remittances to the country of origin. This phenomenon becomes more visible when the deciles are based on migrants alone since the absolute income in the first decile is lower than it is when defining the deciles in the full population. Additionally, we defined the income deciles for men and women separately and within each birth cohort, see Supplementary figure S3. This provides us with a better indicator of individual relative income position though a worse indicator of material resources since members of the same decile no longer have the same absolute income. The patterns are similar to those presented in figure 1.

Administrative registers provide high-quality information on the total population. However, registers do not contain information on several potentially important factors including ethnicity, language proficiency, experiences of discrimination, unregistered incomes or remittances. Alternative data sources, such as surveys, could therefore provide important supplementary analyses, though these are more likely to suffer from other issues, such as small samples, selection bias, various forms of reporting bias and attrition during follow-up.

According to the ‘salmon bias’ hypothesis, mortality rates among migrants may be suppressed if migrants leave the country of destination without de-registering, for example to seek out healthcare or to be with family in the country of origin. If this is more common at lower income levels, this could contribute to the observation that the income gradient in mortality is less pronounced among migrants. In our main analysis, we censor individuals that are registered as emigrated. We conducted a sensitivity analyses that further restricted the sample to individuals that received some form of individual income in the year prior to death. This excluded 127 032 (2.26%) individuals from the sample, but did not change the observed patterns. Details are provided in the Supplementary tables S6 and S7 and figures S4 and S5. Additionally, two recent studies on Swedish register data have indicated that the salmon bias cannot explain the large differences in mortality between migrants and natives in Sweden.^39^^,^^40^

It is important to note that the origin groups we investigate are broad and heterogenous. Sweden is a small country that has only recently received a substantial number of distant migrants, many who are young and experience low mortality risks. Country of birth was divided into regions of origin in the data available to us and a finer country or region grouping provides smaller cells and more volatile estimates (see Supplementary figure S6 for estimates using a more detailed grouping of countries). Since we did not have access to the specific country of birth, we were also unable to use alternative groupings, for example distinguishing migrants from EU countries or by GDP or HDI in the country of origin. Alternative or more detailed categories would require additional data points but also a more careful consideration of specific factors and mechanisms pertaining to specific migrant groups.

## Conclusions

We compare the income gradient in mortality among natives and migrants in Sweden and find that the gradient is less steep among migrants compared with natives and among distant migrants, migrants that arrived as adults and migrants that received their education in Sweden as opposed to abroad. These findings may also provide indirect insights into the life-course processes that generate socio-economic inequalities in health. Given their longitudinal and multidirectional nature, the processes that give rise to social gradients in health are difficult to observe in a single analysis. Our results provide indirect support for the existence of such generative processes by comparing groups for which these processes have been disrupted to groups for whom they have played out.

## Supplementary Material

ckad051_Supplementary_Data

## Data Availability

Data may be obtained from a third party and are not publicly available. The data used in this study were collected from Swedish administrative registers. These data can be requested for research use from Statistics Sweden and the National Board of Health and Welfare.
