# Supplementary material for: Disruption and selection: the income gradient in mortality among natives and migrants in Sweden
Source: Eur J Public Health. 2023 Apr 6;33(3):372–7. doi: 10.1093/eurpub/ckad051 (PMC10234655; doi:10.1093/eurpub/ckad051)

Disruption and selection: The income gradient in mortality among natives and migrants in Sweden

Supplementary materials

Table S1. Classification of country of education

The classification is based on the variable KällKod in the Swedish educational register. It documents the source of the information recorded in the register. Based on the source we coded the individual as educated in Sweden, abroad or unknown. In the analyses, individuals where the source of information was unknown were classified as being educated outside of Sweden. This was done because the collection of information from educational institutions in Sweden is automatic and missing data on education can therefore be interpreted as the person not being educated in Sweden.

Code	Description (Swedish)	Coding
***	Uppgift saknas	Unknown
240	Svenska för invandrare (SFI)	Abroad
260	Register över sökande till KomVux	Sweden
265	Migrationsverket, Registret för Migrations- och asylstatistik (MOA)	Abroad
270	Arbetsförmedlingen, etableringsersättning till nyanlända invandrare	Abroad
280	Arbetsförmedlingen	Sweden
300	Folk- och bostadsräkningen (FoB 70)	Sweden
320	Migrationsverket/Statens invandrarverk (SIV)	Abroad
340	Privattjänstemän	Sweden
360	Primärkommunalt anställda	Sweden
380	Landstingsanställda	Sweden
400	Statsanställda	Sweden
420	Centrala studiestödsnämnden (CSN)	Sweden
430	Arbetsförmedlingen	Sweden
440	Undersökningen om levnadsförhållanden (ULF)	Sweden
460	Arbetskraftsundersökningarna (AKU)	Sweden
465	Registret för Migrations- och asylstatistik (MOA), SSYK-uppgift	Abroad
470	Sökande till högskolan	Sweden
472	Arbetskraftsundersökningarna (AKU)	Sweden
480	Utbildning i annat land än Sverige (enkätundersökning)	Abroad
481	Utbildning i annat land än Sverige (enkätundersökning)	Abroad
482	Utbildning i annat land än Sverige (enkätundersökning)	Abroad
485	Enkät till högutbildade utrikes födda	Abroad
490	Undersökningen om levnadsförhållanden (ULF)	Sweden
500	Lärarregistret	Sweden
520	Lärarregistret	Sweden
525	Gymnasieintyg	Sweden
530	Komvux	Sweden
535	Samlat betygsdokument	Sweden
536	Samlat betygsdokument kombinerat med Komvux	Sweden
540	Avklarade poäng i högskolan	Sweden
560	Sökande/intagna till gymnasieskolan tidigare utbildning	Sweden
580	Folk- och bostadsräkningen (FoB 90)	Sweden
600	Behörighetsgivande utbildning till högskolan	Sweden
620	Arbetsmarknadsutbildningar	Sweden
640	Komvux-kurser	Sweden
655	Folkhögskolan, gymnasiebehörighet	Sweden
660	Komvux	Sweden
665	Folkhögskolan, behörighet till YH-utbildning	Sweden
670	Komvux, gymnasieexaminerade	Sweden
673	Folkhögskolan (grundläggande behörighet)	Sweden
680	Socialstyrelsen	Sweden
685	Isländska centralbyrån	Abroad
690	Statistikcentralen, Finland	Abroad
700	Utl utbildning, högskolornas PA-system	Abroad

(Table continues on next page)

Table S1. Classification of education (continued)

Code	Description (Swedish)	Coding
702	UHR, utländsk gymnasieutbildning	Abroad
705	Socialstyrelsen	Sweden
706	Socialstyrelsen (specialistsjuksköterskor)	Sweden
707	YH-myndigheten/Universitets-och högskolerådet (UHR), utländska yrkeshögskoleutbildningar	Abroad
708	Jordbruksverket	Sweden
710	Högskoleverket (HsV)/Universitets-och högskolerådet (UHR), utländska högskoleutbildningar	Abroad
712	Skolverket, utländska lärarutbildningar	Abroad
715	Försvarsmakten	Sweden
717	Tullverket	Sweden
718	Kriminalvården	Sweden
720	Registret över examinerade från viss gymnasial och eftergymnasial utbildning (EXTAS)	Sweden
730	Kontakttolkutbildning	Sweden
740	Grundexamen till forskare	Sweden
750	Enkät UF-personal, utländsk forskarutbildning (intermittent)	Abroad
760	Kombinationsutbildningar	Sweden
775	Gymnasieskolan, slutbetyg i årskurs 9	Sweden
780	Grundskola, årskurs 9-registret	Sweden
781	Grundskola, specialskolan	Sweden
800	Gymnasieskolans specialkurser	Sweden
810	KKV-utbildning	Sweden
815	Kompletterande utbildning (KU)	Sweden
816	Utlandsskolor; grundskola och gymnasium	Abroad
820	Gymnasieskolans avgångsregister	Sweden
825	Ackumulerat register över avgångna från gymnasieskolan	Sweden
828	Universitets- och högskoleregistret (HREG), basår	Sweden
830	Universitets- och högskoleregistret (HREG), avklarade poäng i högskolan	Sweden
835	Avgångna från kvalificerad yrkesutbildning (KY)/yrkeshögskoleutbildning (YH)	Sweden
837	Försvarshögskolan	Sweden
840	Universitets- och högskoleregistret (HREG)	Sweden
860	Forskarregistret	Sweden
880	Egen uppgift	Unknown
900	Folk- och bostadsräkningen (FoB 90)	Sweden
920	Folk- och bostadsräkningen (FoB 90)	Sweden
940	Folk- och bostadsräkningen (FoB 90)	Sweden
960	Folk- och bostadsräkningen (FoB 90)	Sweden
980	Egen uppgift	Unknown

Table S2. Coefficients used to estimate mortality risks presented in Figure 2. Poisson regression estimating the association between income and mortality with interaction terms between income and country of origin, age at arrival and country of education among foreign-born individuals aged 30-79 in Sweden, 2015-2017. Adjusted for age and sex.

Covariate	Coef.	SE	p
Log income	-0.580	0.030	0.000
Country of birth: Nordic (ref.)	0		
Country of birth: Europe	-0.590	0.193	0.002
Country of birth: Europe × Log income	0.036	0.026	0.159
Country of birth: Other	-0.747	0.141	0.000
Country of birth: Other × Log income	0.058	0.019	0.002
Age at arrival: 0-6 (ref.)	0		
Age at arrival: 7-17	-0.387	0.308	0.208
Age at arrival: 7-17 × Log income	0.024	0.041	0.554
Age at arrival: 18+	-1.933	0.246	0.000
Age at arrival: 18+ × Log income	0.214	0.032	0,000
Country of education: Sweden (ref.)	0		
Country of education: Other	-1.999	0.142	0.000
Country of education: Other × Log income	0.268	0.020	0.000
Sex: Man (ref.)	0		
Sex: Woman	-0.495	0.017	0.000
Age	0.096	0.007	0.000
Age squared	0.000	0.000	0.262
Intercept	-6.079	0.310	0.000

Note: Number of deaths were defined as the dependent variables and person-years at risk as the offset.

Figure S1. Age adjusted death rate by income decile and by country of birth among migrants aged 30-79 in Sweden, 2015-2017 among men (panel a) and women (panel b). The income deciles are ordered from the bottom incomes (1) to the top incomes (10).

a. Men

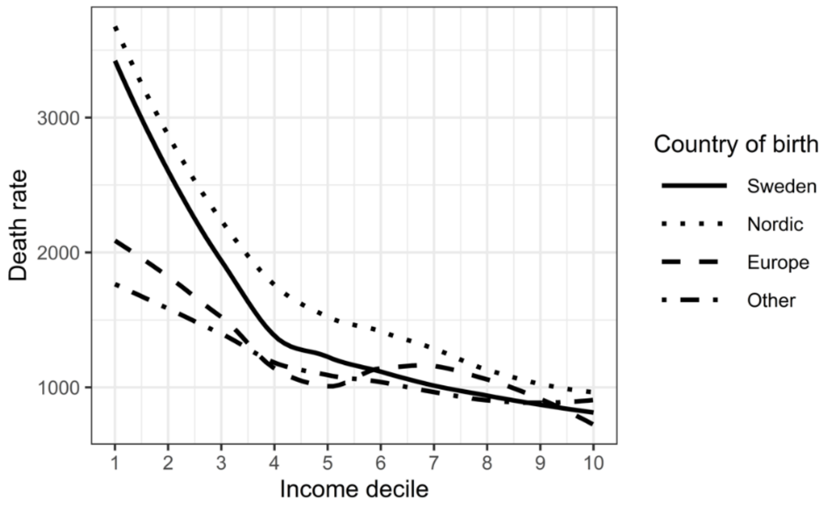

b. Women

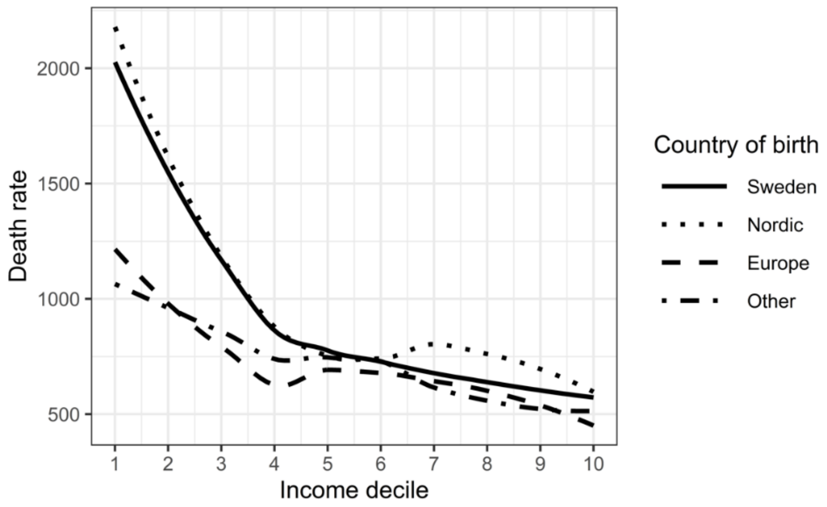

Table S3. Coefficients from a Poisson regression estimating the association between income and mortality with interaction terms between income and country of birth, age at arrival and country of education among foreign-born individuals aged 30-79 in Sweden, 2015-2017. Adjusted for age.

	Men			Women		
Covariate	Coef.	SE	p	Coef.	SE	p
Log income	-0.585	0.034	0.000	-0.564	0.058	0.000
Country of birth: Nordic (ref.)	0			0		
Country of birth: Europe	-0.451	0.234	0.054	-0.843	0.347	0.015
Country of birth: Europe × Log income	0.018	0.031	0.563	0.067	0.047	0.148
Country of birth: Other	-0.740	0.182	0.000	-0.866	0.224	0.000
Country of birth: Other × Log income	0.053	0.024	0.031	0.080	0.030	0.008
Age at arrival: 0-6 (ref.)	0			0		
Age at arrival: 7-17	-0.556	0.364	0.127	0.049	0.582	0.932
Age at arrival: 7-17 × Log income	0.051	0.048	0.289	-0.040	0.077	0.605
Age at arrival: 18+	-1.965	0.291	0.000	-1.806	0.470	0.000
Age at arrival: 18+ × Log income	0.222	0.038	0.000	0.193	0.061	0.002
Country of education: Sweden (ref.)	0			0		
Country of education: Other	-2.212	0.184	0.000	-1.649	0.226	0.000
Country of education: Other × Log income	0.291	0.025	0.000	0.228	0.031	0.000
Age	0.095	0.009	0.000	0.099	0.012	0.000
Age squared	0.000	0.000	0.393	0.000	0.000	0.542
Intercept	-5.943	0.379	0.000	-6.864	0.558	0.000

Table S4. VIF statistics.

Covariate	GVIF
Income	1.064615
Country of origin	1.274452
Age group at arrival	1.309047
Country of education	1.258714
Sex	1.006291
Age	1.314137

Note: The estimates were obtained from an OLS model where death was the outcome. Higher values indicate stronger correlations between covariates. A value of 1 indicates no correlation between a covariate and any other covariates. Values greater than 5 indicate that the results of the regression may be unreliable due to multicollinearity. We used an OLS model to obtain VIF statistics, since these can not be calculated from Poisson models. However, the choice of model does not influence the correlation between the covariates.

Table S5. Median income and proportion of the population in different origin groups by income decile when defining the deciles using either the income distribution in full population or among migrants only. The income deciles are ordered from the bottom incomes (1) to the top incomes (10).

	Median income (1000 SEK) by decile									
Population	1	2	3	4	5	6	7	8	9	10
Full population	105	149	187	218	246	274	305	344	401	543
Migrants	51	104	129	152	180	209	240	276	327	437
	Proportion of the population (%) by deciles, defined in the full population									
Country of origin	1	2	3	4	5	6	7	8	9	10
Sweden	7	9	10	10	10	11	11	11	11	11
Nordic	14	13	11	10	9	9	9	9	9	8
Europe	18	11	10	9	8	8	8	8	9	9
Other	29	16	12	10	8	7	6	5	4	3
	Proportion of the population (%) by deciles, defined among migrants only									
Country of origin	1	2	3	4	5	6	7	8	9	10
Sweden	-	-	-	-	-	-	-	-	-	-
Nordic	6	5	7	9	9	10	11	12	14	17
Europe	10	5	6	8	8	9	10	11	14	19
Other	11	12	11	11	10	10	10	9	9	7

Figure S2. Age adjusted death rate by income decile by country of birth among migrants aged 30-79 in Sweden, 2015-2017. The income deciles are ordered from the bottom incomes (1) to the top incomes (10). The income deciles are defined by the income distribution among migrants.

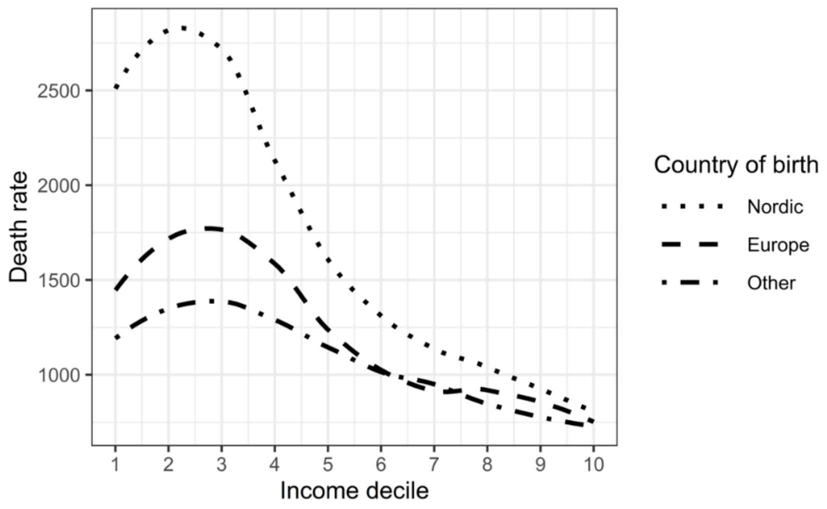

Figure S3. Age adjusted death rate by income decile by country of birth among migrants aged 30-79 in Sweden, 2015-2017. The income deciles are ordered from the bottom incomes (1) to the top incomes (10). The income deciles are defined within each birth cohort and separately by sex.

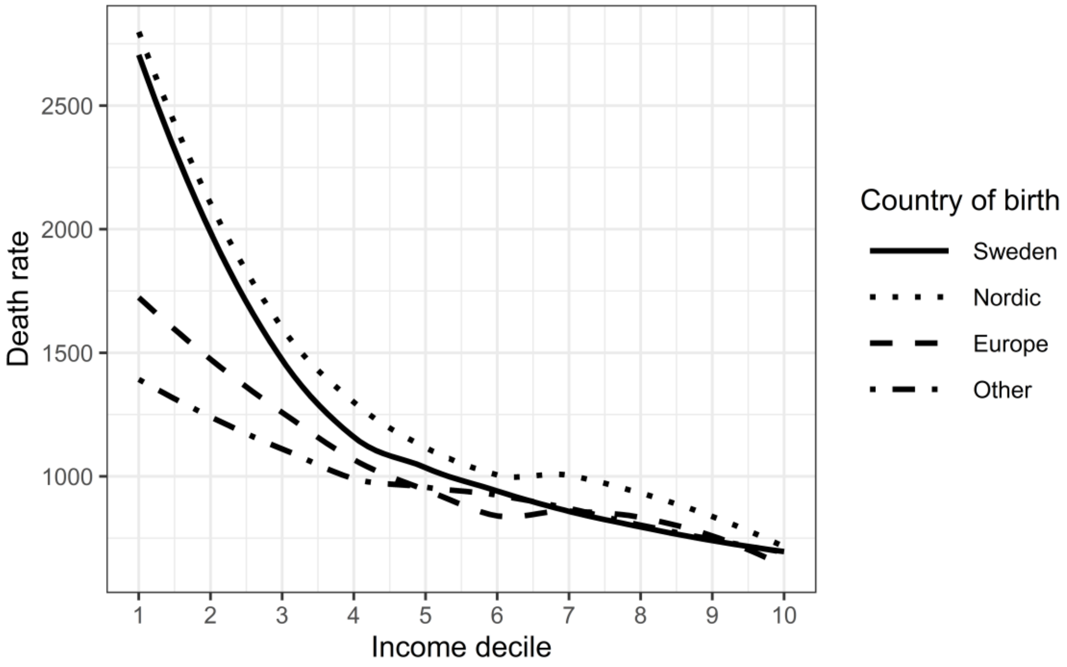

Table S6. The number of persons censored due to not having any registered individual income during 2014, 2015 and 2016 by country of birth.

Country of birth	Retained (n)	Censored (n)	Censored (%)
Sweden	4529165	80020	1.74
Nordic	170863	5922	3.35
Europe	77117	5065	6.16
Other	707296	36025	4.85
Total	5484441	127032	2.26

Figure S4. The income gradient in age adjusted mortality by country of birth among migrants aged 30-79 in Sweden, 2015-2017. The income deciles are ordered from the bottom incomes (1) to the top incomes (10). The sample is restricted to those that had some form of individual income.

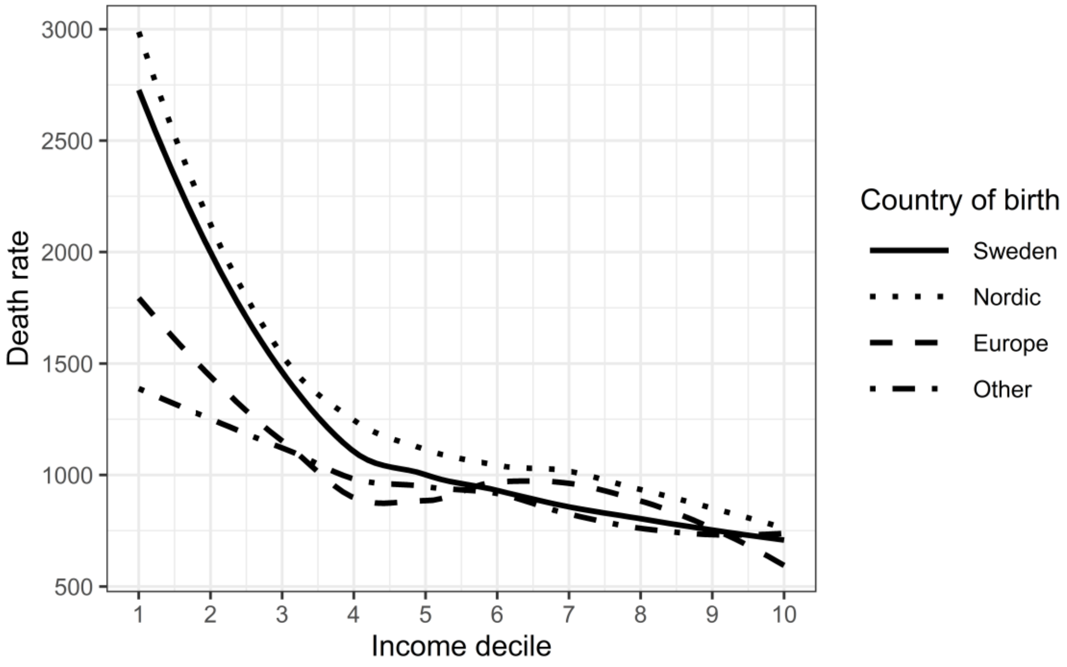

Table S7. Coefficients from a Poisson regression estimating the association between income and mortality with interaction terms between income and country of origin, age at arrival and country of education among foreign-born individuals aged 30-79 in Sweden, 2015-2017. Adjusted for age. The sample is restricted to those that had some form of individual income.

Covariate	Coef.	SE	p
Log income	-0.649	0.031	0.000
Country of origin: Nordic (ref.)	0		
Country of origin: Europe	-0.959	0.247	0.002
Country of origin: Europe × Log income	0.085	0.033	0.009
Country of origin: Other	-1.030	0.168	0.000
Country of origin: Other × Log income	0.095	0.022	0.002
Age at arrival: 0-6 (ref.)	0		
Age at arrival: 7-17	-0.032	0.333	0.923
Age at arrival: 7-17 × Log income	-0.022	0.044	0.623
Age at arrival: 18+	-1.682	0.272	0.000
Age at arrival: 18+ × Log income	0.182	0.036	0,000
Country of education: Sweden (ref.)	0		
Country of education: Other	-1.915	0.173	0.000
Country of education: Other × Log income	0.255	0.024	0.000
Sex: Man (ref.)	0		
Sex: Woman	-0.509	0.017	0.000
Age	0.101	0.008	0.000
Age squared	0.000	0.000	0.759
Intercept	-5.660	0.327	0.000

Figure S5. Estimated mortality risk by log income and (a) country of birth, (b) age at arrival and (c) country of education among migrants aged 30-79 in Sweden, 2015-2017.The sample is restricted to those that had some form of individual income. Based on the coefficients presented in Table S7.

a. Country of birth

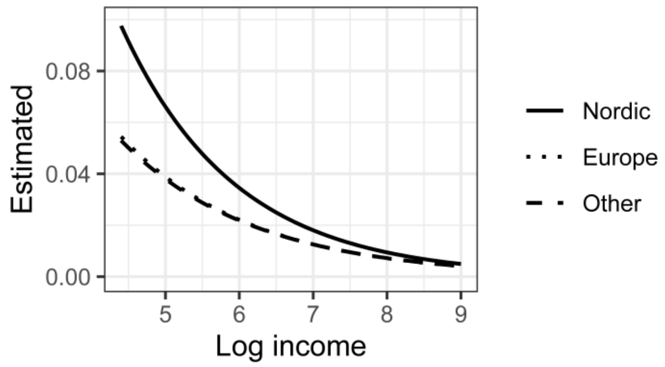

b. Age at arrival

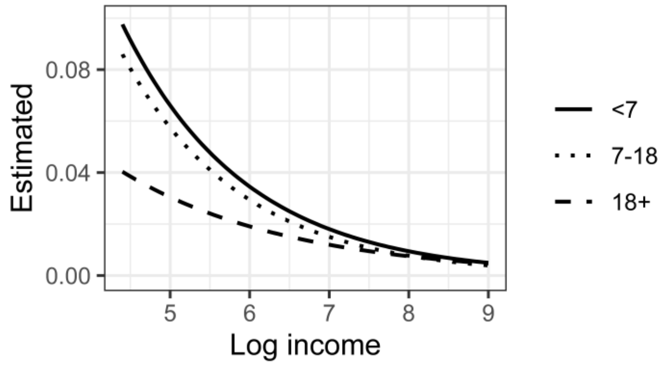

c. Country of education

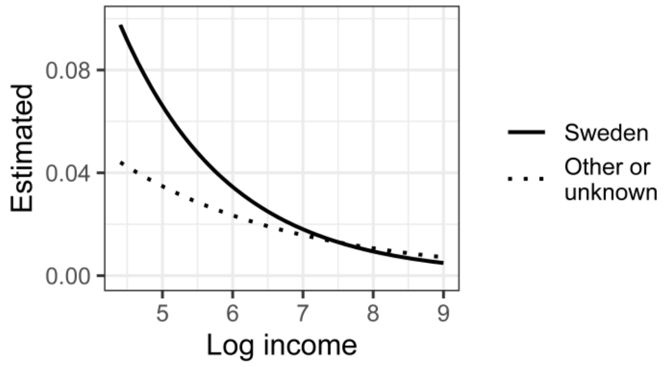

Figure S6. The income gradient in age adjusted mortality by country of birth in seven categories among migrants aged 30-79 in Sweden, 2015-2017. The income deciles are ordered from the bottom incomes (1) to the top incomes (10).

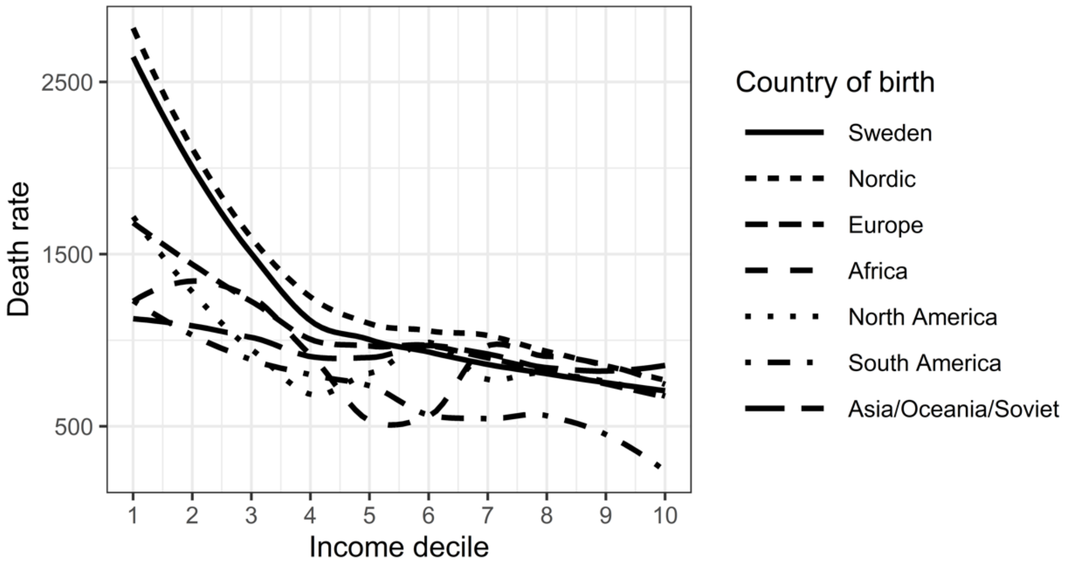

Supplement: ckad051_Supplementary_Data [file ckad051_supplementary_data.pdf]
